# A Quick Guide to Small-Molecule Inhibitors of Eukaryotic Protein Synthesis

**DOI:** 10.1134/S0006297920110097

**Published:** 2020-11-26

**Authors:** S. E. Dmitriev, D. O. Vladimirov, K. A. Lashkevich

**Affiliations:** 1 Belozersky Institute of Physico-Chemical Biology, Lomonosov Moscow State University, 119234 Moscow, Russia; 2 Faculty of Bioengineering and Bioinformatics, Lomonosov Moscow State University, 119234 Moscow, Russia; 3 Engelhardt Institute of Molecular Biology, Russian Academy of Sciences, 119991 Moscow, Russia

**Keywords:** small-molecule drugs, 40S and 60S ribosomal subunits, 4E-BP1, eIF2α phosphorylation, ribotoxic stress, cycloheximide, harringtonine, trichothecene mycotoxins, aminoglycosides, rapamycin

## Abstract

Eukaryotic ribosome and cap-dependent translation are attractive targets in the antitumor, antiviral, anti-inflammatory, and antiparasitic therapies. Currently, a broad array of small-molecule drugs is known that specifically inhibit protein synthesis in eukaryotic cells. Many of them are well-studied ribosome-targeting antibiotics that block translocation, the peptidyl transferase center or the polypeptide exit tunnel, modulate the binding of translation machinery components to the ribosome, and induce miscoding, premature termination or stop codon readthrough. Such inhibitors are widely used as anticancer, anthelmintic and antifungal agents in medicine, as well as fungicides in agriculture. Chemicals that affect the accuracy of stop codon recognition are promising drugs for the nonsense suppression therapy of hereditary diseases and restoration of tumor suppressor function in cancer cells. Other compounds inhibit aminoacyl-tRNA synthetases, translation factors, and components of translation-associated signaling pathways, including mTOR kinase. Some of them have antidepressant, immunosuppressive and geroprotective properties. Translation inhibitors are also used in research for gene expression analysis by ribosome profiling, as well as in cell culture techniques. In this article, we review well-studied and less known inhibitors of eukaryotic protein synthesis (with the exception of mitochondrial and plastid translation) classified by their targets and briefly describe the action mechanisms of these compounds. We also present a continuously updated database (http://eupsic.belozersky.msu.ru/) that currently contains information on 370 inhibitors of eukaryotic protein synthesis.

## INTRODUCTION

Eukaryotic translation machinery has several specific features, both in the structure of its components and mechanisms of translation cycle [1-3]. Despite conservation of the functional core, eukaryotic ribosome significantly differs from the bacterial one in structural details, having much in common with the archaeal ribosome. It also contains a number of eukaryote-specific elements, including additional rRNA segments, proteins, and protein regions [2, 3]. In the course of evolution, eukaryotes have developed unique features of translation initiation, termination, and ribosome recycling [1, 4-6]. The most prominent one is the cap-dependent ribosomal scanning, which occurs during translation initiation and involves loading of the 40S ribosomal subunit near the 5′-end of mRNA (that usually contains the m^7^G-cap) and its directional movement towards the 3′-end until the start codon [1, 5].

The presence of both conserved and specific features explains the fact that compounds suppressing protein biosynthesis in eukaryotic cells include both universal ribosome-targeting antibiotics (active in organisms from all kingdoms of life) and eukaryote-specific inhibitors of ribosomes or other components of the translational apparatus. These compounds interact with different functional sites: the peptidyl transferase center (PTC), the E-site, the polypeptide exit tunnel (PET), or the GTPase-activating center (GAC) of the 60S ribosomal subunit; the decoding center (DC) or other sites of the 40S subunit; the binding sites of translation factors or translation-related proteins themselves, etc. [7-9].

Beside acting on specific targets and having different mechanisms of action, translation inhibitors may also differ in their effect on polysomes, which can be easily observed in direct experiments. Compounds that block initiation, but not elongation, usually disassemble polysomes. Elongation inhibitors can either disassemble or stabilize polysomes, depending on whether they are able to act on internal ribosomes in the polysome or only on the *de novo* initiating ribosomes (see below). The latter statement is not obvious and often causes confusion, so some compounds acting at the elongation stage (for example, harringtonine and lactimidomycin) are sometimes called initiation inhibitors in the literature. Termination inhibitors can increase the number of ribosomes in a polysome; however, compounds that cause the stop codon readthrough usually do not modify the polysome profile. The same is true for the compounds causing miscoding; they decrease the fidelity of protein synthesis, but generally do not affect the polysomes. Premature termination inducers (e.g., puromycin) disassembles polysomes. This issue is complicated by the fact that some inhibitors exhibit the concentration-dependent effects or trigger the ribotoxic or other types of stress in living cells, which might change the pattern of cell response to the inhibitor over time.

Here, we compiled a panel of small-molecule inhibitors of eukaryotic translation and listed them in the tables with minimalistic comments. The most studied inhibitors are described in detail in the text of the article (see also figure). The additional information can be found in a constantly updated database (http://eupsic.belozersky.msu.ru/) at the Belozersky Institute of Physico-Chemical Biology, Moscow State University. Due to the limited space, we did not discuss inhibitors of mitochondrial and plastid translation, since ribosomes of these organelles belong to the bacterial type. We also omitted protein and peptide inhibitors of translation (such as ricin or diphtheria toxin) despite their importance and widespread usage.

**Figure.**
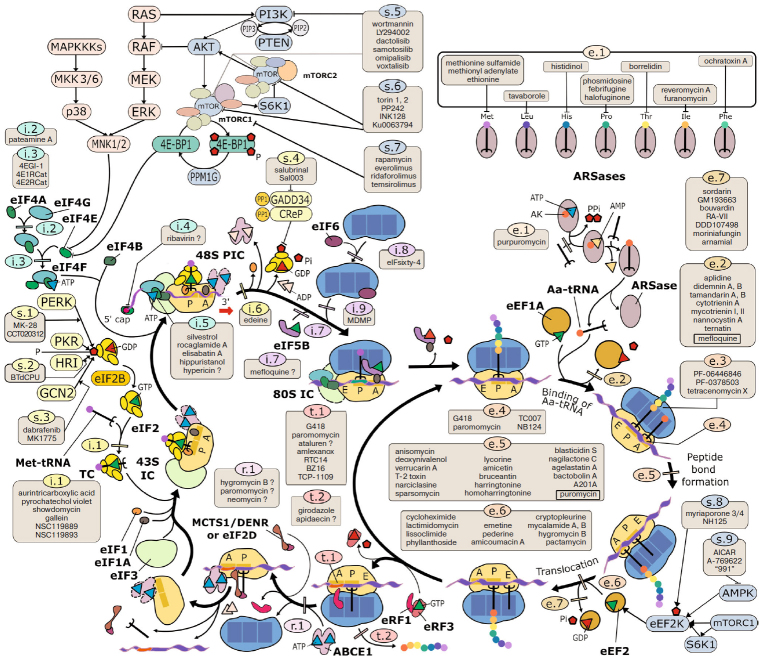
Eukaryotic translation cycle, selected regulatory pathways, and the most commonly used and well-characterized inhibitors of protein synthesis. The inhibitors are grouped according to the translation cycle stages, in which their targets are involved. Translation initiation: i.1, Met-tRNA_i_ binding to eIF2 and formation of the eIF2/Met-tRNA_i_/GTP ternary complex (TC); i.2, eIF4A binging to eIF4G; i.3, eIF4E binging to eIF4G; i.4, eIF4E binging to the m^7^G-capped mRNA 5′-end; i.5, eIF4A helicase activity during eIF4F binding to the mRNA and subsequent ribosome scanning; i.6, AUG codon recognition during scanning; i.7, eIF5B interaction with the 60S subunit; i.8, eIF6 interaction with the 60S subunit; i.9, 60S subunit recruitment to the 48S preinitiation complex (48S PIC) and formation of the 80S initiation complex (80S IC). Elongation and accompanying reactions: e.1, tRNA aminoacylation; e.2, eEF1A/GDP dissociation after delivery of aminoacyl-tRNA (Aa-tRNA); e.3, polypeptide progression in the ribosomal tunnel; e.4, tRNA accommodation/decoding; e.5, peptidyl transferase reaction (combined with the preceding stages of Aa-tRNA binding and accommodation); e.6, translocation; e.7, eEF2/GDP dissociation after translocation. Termination: t.1, stop codon recognition; t.2, peptidyl-tRNA hydrolysis. Recycling: r.1, 60S subunit dissociation. Modulators of signaling cascades: s.1-s.3, activators of eIF2 kinases; s.4, eIF2 phosphatase inhibitors; s.5, PI3K inhibitors; s.6, mTOR active site inhibitors; s.7, allosteric inhibitors of mTOR in the mTORC1 complex. Inhibitors with different mechanisms of actions affecting the same stage are shown in frames.

## INHIBITORS OF EUKARYOTIC RIBOSOME

There are several types of ribosome inhibitors common to all kingdoms of life. Most of them target conserved stages of the elongation cycle, such as ligand binding, transpeptidation, and translocation, and will be discussed in the first section of our review (Table 1). Inhibitors active toward all ribosome types will be hereafter called universal inhibitors. Otherwise, we will use the terms “eukaryote-specific” or “archaea- and eukaryote (AE)-specific”. The specificity is usually determined by subtle differences in the structure of the binding site. Structural studies have shown that substitution of a single nucleotide in rRNA or a difference in one amino acid residue in a ribosomal protein might be sufficient to change ribosome configuration enough to preclude the binding of the inhibitor. Many of the inhibitors had been identified back in the 1960-70s due to the efforts of several groups of scientists, among which D. Vázquez, A. Jiménez, and S. Pestka should be specially recognized. In our review, we describe the results of studies conducted since the late 1960s, while earlier findings and the history of inhibitors that had been discovered more than half a century ago can be found in the classic reviews of the above-mentioned authors [10-13].

**Table 1.** Small-molecule inhibitors of eukaryotic ribosome

Name	Class, group of chemical substances	Specificity (B, A, E)^#^	Interacting ribosomal subunit	Binding site^##^	Stage of the translation cycle^###^	Effect on polysomes^####^	Mechanism of action
Anisomycin/flagecidin	pyrrolidine, anisomycin group	A, E	60S	PTC (A)	E	stab	PTC inhibitor
Deacetylanisomycin	– // –	E	60S	PTC (A)	E	stab?	– // –
Preussin/L-657,398	– // –	E	60S	PTC (A)?	E?		– // –
Calonectrin	trichothecene A		60S?	PTC (A)?	E	stab/dis	– // –
Neosolaniol	– // –	E	60S?	PTC (A)?	E?	?	– // – ?
Scirpentriol	– // –	B, A, E	60S	PTC (A)	E	dis	– // –
Diacetoxyscirpenol/anguidine	– // –	B, A, E	60S	PTC (A)	E	dis	– // –
T-2 toxin	– // –	E	60S	PTC (A)	E	dis	– // –
T-2 triol	– // –	E	60S?	PTC (A)?	E?	?	– // – ?
Trichodermin	– // –	E	60S	PTC	E	stab/dis	– // –
Trichodermol	– // –	E	60S	PTC	E	stab	– // –
Diacetylverrucarol	– // –	E	60S?	PTC (A)?	E?	?	– // – ?
Trichothecolone	– // –	B, A, E	60S?	PTC (A)?	E?	stab/dis	– // – ?
Trichothecin	trichothecene B	E	60S	PTC (A)?	E	stab/dis	– // –
Fusarenone X	– // –	E	60S	PTC	E	stab	– // –
Vomitoxin/deoxynivalenol	– // –	E	60S	PTC (A)	E	dis	– // –
Nivalenol	– // –	E	60S?	PTC (A)?	E?	dis	– // – ?
Crotocin	trichothecene C	E	60S	PTC (A)	E, T?	stab	– // – ?
Satratoxin G	trichothecene D	E	60S	PTC (A)	E?	dis	– // – ?
Roridin A	– // –	E	60S?	PTC (A)?	E?	?	– // – ?
Myrothecin A	– // –	E	60S?	PTC (A)?	E?	?	– // – ?
Verrucarin A/muconomycin A	trichothecene D, muconomycin	E	60S	PTC (A)	E	stab	– // –
Muconomycin B	– // –	E	60S?	PTC (A)?	E?	stab	– // –
Narciclasine	tetraheterocyclic alkaloid	B, A, E	60S	PTC (A)	E	stab	– // –
Isonarciclasine	– // –	B, A, E	60S?	PTC (A)?	E	stab	– // –
Lycorine	– // –	B, A, E	60S	PTC (A)	E	?	– // –
Pseudolycorine	– // –	B, A, E	60S	PTC (A)	E	?	– // –
Haemanthamine	– // –	A, E	60S	PTC (A)	E	stab	– // –
Haemanthidine	– // –	A, E	60S	PTC (A)	E	(stab)	– // –
Bulbispermine/hamayne	– // –	E	60S	PTC (A)	E	(stab)	– // –
Pretazettine	– // –	E	60S	PTC (A)	E	stab	– // –
Jonquailine	– // –	E?	60S?	PTC (A)?	E?	?	– // – ?
Crinamine	– // –	B, A, E	60S	PTC (A)	E	(stab)	– // –
Agelastatin A	heterocyclic alkaloid	E	60S	PTC (A)	E	?	– // –
Cephalotaxine	heterocyclic alkaloid, cephalotaxine group	(E)	60S	PTC (A)?	(E)	(stab)	– // – (weak)
Harringtonine	– // –	A, E	60S	PTC (A)	E	dis	– // –
Homoharringtonine/omacetaxine mepesuccinate	– // –	A, E	60S	PTC (A)	E	dis	– // –
Nagilactone C	diterpenoid	E	60S	PTC (A)	E	dis	– // –
Nagilactone E	– // –	E	60S?	PTC (A)?	E	?	– // – ?
Bruceantin	quassinoid	A, E	60S	PTC (A)	E	?	– // –
Grandilactone A	– // –	E	60S?	PTC (A)?	E	?	– // –
Brusatol	– // –	E	60S?	PTC (A)	E?	?	– // – ?
Holacanthone	– // –	E?	60S	PTC (A)	?	?	– // –
Baccharinol	– // –	E	60S?	PTC (A)?	E?	?	– // – ?
Ailanthinone	– // –	E	60S?	PTC (A)?	E?	?	– // – ?
Quassin	– // –	E?	60S?	PTC (A)?	E?	?	– // – ?
Sparsomycin	pyrimidone, sparsomycin group	B, A, E	60S	PTC (A, P)	E	stab	– // –
Deshydroxysparsomycin	– // –	E	60S?	PTC (A, P)?	E?	?	– // – ?
Octylsparsomycin	– // –	E	60S?	PTC (A, P)?	E?	?	– // – ?
Phenol-alanine sparsomycin	– // –	B, A, E	60S?	PTC (A, P)?	E?	?	– // – ?
MDL 20828	– // –	E	60S?	PTC (A, P)?	E?	?	– // – ?
Anthelmycin/hikizimycin	nucleoside, pyrimidone	B, A, E	60S	PTC (P)	E	(stab)	– // –
Blasticidin S	nucleoside, blasticidin group	B, A, E	60S	PTC (P)	E	?	– // –
Gougerotin	– // –	B, A, E	60S	PTC	E	dis	– // –
Bagougeramine A	– // –	B, A, E?	60S?	PTC?	E?	?	– // – ?
Amicetin	– // –	B, A, E	60S	PTC	E	stab	– // –
Bamicetin	– // –	B, A, E	60S	PTC	E	?	– // –
Mildiomycin	– // –	B, A, E	60S	PTC	E	?	– // –
Plicacetine	– // –	B, A, E	60S?	PTC?	E?	?	– // – (weak)
Arginomycin	– // –	B, A, E	60S?	PTC?	E?	?	– // – ?
Puromycin	aminoacyl-nucleoside	B, A, E	60S	A	E	dis	indices premature termination
A201A	– // –	B, A, E	60S	A	E		PTC inhibitor
Bactobolin	isocoumarin	B, A, E?	60S	PTC (P)	E	?	– // –
Actinobolin	– // –	B, A, E	60S	PTC	E	?	– // –
Amicoumacin A	– // –	B, A, E	40S	E-site	E	?	translocation inhibitor
Baciphelacin	– // –	B, A, E	?	?	I?	?	?
Oosponol	– // –	B, A, E?	?	?	?	?	?
AHR-1911	thiopseudourea	B, A, E	60S?	PTC?	E		PTC inhibitor
Tenuazonic acid	pyrroline	E	60S?	PTC (A, P)	?	stab	– // –
Bottromycin A2	cyclic peptide	B, A, E?	60S?	PTC?	E?	stab?	– // – ?
Griseoviridin	cyclodepsipeptide	B, A, E	60S	PTC	E	?	– // – ?
Cyclopiazonic acid	ergoline alkaloid	B, A, E	60S?	?	E	?	– // – ?
PF-06446846/PF846		B, A, E	60S	PET	E	?	alters path of nascent peptide, blocks translocation
PF-06378503/PF8503		E	60S?	PET?	E	?	– // – ?
Tetracenomycin X	aromatic polyketide, tetracenomycin group	B, A, E	60S	PET	E?	?	blocks PET, hinder peptide progression
Tetracycline Col-3	aromatic polyketide, tetracycline group	B, A, E	40S, 60S	PET and other	E?	?	?
Doxycycline	– // –	B, A, E	40S, 60S	PET and other	E?	?	?
Tigecycline	– // –	B, A, E	40S? 60S?	?	E	?	inhibits Aa-tRNA binding?
Minocycline	– // –	B, A, E	40S? 60S?	?	E?	?	?
Doxorubicin	aromatic polyketide, anthracycline group	E?	?	?	T	?	promotes stop-codon readthrough
Cycloheximide/naramycin A/actidion	glutarimide	E	60S	E-site	E	stab	translocation inhibitor
Naramycin B	– // –	E	60S?	E-site?	?	?	– // – ?
Acetoxycycloheximide	– // –	E	60S	E-site?	E	stab	– // – ?
Streptimidone	– // –	E	60S	E-site?	E	?	– // – ?
Streptovitacin A	– // –	E	60S	E-site?	E?	stab	– // – ?
Actiphenol	– // –	E	60S?	E-site?	E?	?	– // – ?
Lactimidomycin	– // –	E	60S	E-site	E	dis	– // –
Isomigrastatin	– // –	E	60S?	E-site?	E?	?	– // – ?
ECA-LTM	– // –	E	60S?	E-site?	E?	?	– // – ?
Streptoglutarimide H	– // –	B, A, E	60S?	E-site?	E?	?	– // – ?
Chlorolissoclimide	labdane diterpenoid, lissoclimide group	E	60S	E-site	E	stab	– // –
Lissoclimide C45	– // –	E	60S	E-site	E	stab	– // – ?
Haterumaimides Q	– // –	E	60S?	E-site?	E	stab	– // –
Phyllanthoside	glycoside	E	60S	E-site	E	dis	– // –
S3′-desacetyl phyllanthoside	– // –	E	60S?	E-site?	?	?	– // – ?
Pederin	polyketide, pederin group	E	60	E-site	E	stab	– // –
Psymberin/irciniastatin A	– // –	E	60S	E-site	E	?	– // –
Theopederin B	– // –	A, E	60S	E-site	E	?	– // –
Mycalamide B	– // –	B, A, E	60S	E-site	E	dis	– // –
Onnamide A	– // –	A? E	60S	E-site	E	?	– // –
Tedanolide	polyketide, tedanolide group	A? E	60S	E-site	E	?	– // –
13-deoxytedanolide	– // –	A, E	60S	E-site	E	?	– // –
Myriaporone 3/4	– // –	A, E	eEF2?	-	E	?	(see Table 3)
Emetine	pyridoisoquinoline alkaloid, emetine group	E	40S	E-site	E	stab	translocation inhibitor
Dehydroemetine	– // –	E	40S?	E-site?	E?	(stab)	– // –
Cephaeline	– // –	E	40S?	E-site?	E?	(stab)	– // –
Cryptopleurine	phenanthroquinolizidine alkaloid, emetine-like	E	40S, 60S?	E-site	E	?	– // –
Tylocrebrine	– // –	B? E	40S?	E-site?	E	?	– // –
Tubulosine	– // –	E	40S, 60S?	E-site?	E	?	– // –
Tylophorine/DCB-3500	– // –	B?, A, E	40S	E-site?	E	stab	– // –
Rac-cryptopleurin	– // –	E	40S?	E-site?	E	?	– // –
YXM-110	– // –	E	40S?	E-site?	E	?	– // –
Pactamycin	aminocyclopentitol, pactamycin group	B, A, E	40S	E-site	E (I?)	(dis)	– // –
de-6-MSA-pactamycin	– // –	B, A, E	40S?	E-site?	E (I?)	?	– // – ?
Zaluzanin C	sesquiterpene lactone	E	?	?	E	?	– // –
Hygromycin B	2-DOS aminoglycoside, noncanonical	B, A, E	40S?	DC	E (R?)	stab	– // –
G418/geneticin	2-DOS aminoglycoside, 4,6-disubstituted	B, E	40S, 60S	DC, PET	E, T	dis	induces miscoding, promotes stop-codon readthrough (HC inhibits translocation)
Gentamicins	– // –	B, E	40S, 60S	DC, PET	E, T	dis	– // –
Tobramycin	– // –	B, E?	40S, 60S?	DC	E, T	dis?	– // –
Amikacin	– // –	B, E	40S	DC	E, T	dis	– // –
Netilmicin	– // –	B, A, E?	40S?	DC?	E?	dis?	– // –
Paromomycin	2-DOS aminoglycoside, 4,5-disubstituted	B, E	40S, 60S	DC	E, T (R?)	dis	– // –
Lividomycin	– // –	B, A, E?	40S?	DC?	E	?	– // –
Neomycin	– // –	B, A, E?	40S?	DC?	E, T (R?)	?	– // –
TC007	– // –	B, A, E	40S, 60S	DC, PET	T	?	– // –
NB74	– // –	B? E	40S	DC	E, T	?	– // –
NB124	– // –	B? E	40S	DC?	E, T	?	– // –
NB156	– // –, NB74 derivative	B?, E	40S	DC	E, T	?	– // –
NB157	– // –, NB124 derivative	B?, E	40S	DC	E, T	?	– // –
Neamine	2-DOS aminoglycoside, 4-monosubstituted	B, E	60S?	DC	E, T	dis?	– // –
Apramycin	– // –	B, E	40S?	DC	E	?	– // – ?
Negamycin	negamycin group	B, A, E	40S	?	T	stab	– // –
3-Epi-deoxynegamycin	– // –	E	?	?	T	?	– // – ?
TCP-1109	– // –	E	40S		T	?	– // –
Ataluren/PTC124	oxadiazoles	E	60S?	A?	T?	?	– // – ?
Amlexanox	benzopyrans	E	?		T		– // –
RTC204		E	?	?	T	?	– // –
RTC219		E	?	?	T	?	– // –
GJ071		E	?	?	T	?	– // –
GJ072		E	?	?	T	?	– // –
GJ103		E	?	?	T	?	– // –
RTC13	thiazolidinone group	E	40S?	?	T	?	– // –
RTC14	– // –	E	?	?	T	?	– // –
BZ6	– // –	E	?	?	T	?	– // –
BZ16	– // –	E	?	?	T	?	– // –
CDX5-1	phthalimide	?	?	?	?	?	enhances induction of stop-codon readthrough by aminoglycosides
RP 49532A/girodazole/girolline		A, E	60S	E-site	T	?	inhibits peptide release
Sanguinamide B	cyclic peptide	B, A, E	40S, 60S	uS17, uL3, uL30, other	?	?	?
Daptomycin	cyclic lipopeptide	B, A?, E	40S	eS19	?	?	?
QL-XII-47/QL47	QL47 group	E	?	?	E?	?	?
YKL-04-085	– // –	E	?	?	E?	?	?
Mefloquine	quinoline	E*	60S	GAC	E	?	impedes accommodation of eEF1A, eEF2 (eIF5B?)
Edeine A		B, A, E	40S	E-site	I	?	affects binding or accommodation of Met-tRNA_i_
MDMP		E	40S? 60S?	?	I	dis	prevents 60S joining
eIFsixty-4		E	60S	?	I	dis	precludes eIF6 binding to 60S

Notes: ^#^ B, bacteria; A, archaea; E, eukaryotes. * Denotes a narrower group of organisms (for example, fungi or protozoa).

^##^ PTC (A), A-site of the PTC; PTC (P), P-site of the PTC; other abbreviation as in the main text.

^###^ I, initiation; E, elongation; T, termination; R, recycling.

^####^ stab, stabilizes polysomes; dis, disassembles polysomes, stab/dis, stabilizes or disassembles polysomes depending on the concentration or other conditions.

“?” means “presumably” (by analogy with a chemically similar compound, based on the information from other organisms or on controversial data); brackets, phenomenon is less pronounced than in other cases; HC, at high concentration.

**Ribosome-targeting elongation inhibitors.** The overwhelming majority of currently known ribosome-targeting inhibitors act at the polypeptide elongation stage. These compounds include inhibitors of peptidyl transferase reaction and translocation, peptide tunnel blockers, inducers of decoding errors (miscoding) and premature termination, as well as some other types of inhibitors with unique mechanisms of action.

*Inhibitors of peptidyl transferase center.* Due to its conservation, the PTC of the large ribosomal subunit is the most vulnerable spot of the “protein-synthesizing machine”. In both pro- and eukaryotes, the largest number of inhibitors, although belonging to different chemical classes and interfering with the ribosome function in different ways, binds at this site (figure, e.5).

Some of these inhibitors interfere with the aminoacyl-tRNA entry or accommodation in the A-site. They include, for example, the classic AE-specific inhibitor anisomycin, which interacts with the A-site and destabilizes aminoacyl-tRNA binding [14-17]. The same site is targeted by the eukaryote-specific trichothecene mycotoxins (T-2 toxin, deoxynivalenol, verrucarin A, and more than three dozen similar compounds with a complex four-membered heterocycle produced by parasitic fungi [14, 17-19]). The tetraheterocyclic plant alkaloids narciclasine, lycorine, haemanthamine [14, 20] and, presumably, their numerous derivatives, such as isonarciclasine, pseudolycorine, pretazettine [21, 22], also bind at the A-site. The same is true for harringtonine [23], an inhibitor widely used in ribosome profiling technique [24], and related homoharringtonine [14, 23, 25, 26]. Homoharringtonine in a form of a semisynthetic drug (omacetaxine mepesuccinate) is among few translation inhibitors approved by both the European Medicines Agency (EMA) and the American Food and Drug Administration (FDA) for the treatment of chronic myeloid leukemia [27]. It has also been considered as a promising drug for the antiviral therapy of COVID-19 [28].

An interesting property of harringtonine and homoharringtonine is that they bind only to vacant 60S/80S particles or ribosome that have just assembled from the subunits and started elongation, so the inhibitors stop elongation immediately (or soon) after the start [23]. At the same time, previously initiated ribosomes continue translation, which results in only one 80S particle remaining on the mRNA at the beginning of the coding region [25, 29]. This makes harringtonine a useful tool for mapping start codons on a genome-wide scale [24].

It should be noted that the inability to bind to actively translating polysomes is not uncommon among elongation inhibitors. When added to the cells, these compounds cause the disassembly of polysomes rather than their stabilization; therefore, they are sometimes erroneously referred to as initiation inhibitors [25, 29]. Some of the above-mentioned trichothecene mycotoxins produce a similar effect on polysomes as harringtonine. Thus, T-2 toxin, verrucarin A, nivalenol, and calonectrin disassemble polysomes, while trichothecin, trichodermin, and scirpentriol, although have the same trichothecene core, stabilize them [19, 30, 31]. The difference in the action was explained by the particular side radicals in the certain positions of the scaffold [19, 30, 32]. In the case of some mycotoxins, the effect may also depend on the drug concentration. For example, diacetoxyscirpenol and fusarenone X, which normally disassemble polysomes, stabilize them when used at a 100-times higher concentration [30, 33]. The ability to bind to the ribosomes with the vacant A-site only and to disassemble polysomes is also typical for some translocation inhibitors, e.g., lactimidomycin [34] (the only case when a mechanism of this phenomenon has been explored, see the text below for the proposed explanation).

The A-site of the PTC is also targeted by other chemicals, whose structure is principally different from the structure of the above inhibitors. They are natural compounds nagilactone C [14, 35] and agelastatin A [36], as well as bruceantin (a member of a wide class of quassinoids, which includes many potential anticancer drugs) [26, 37, 38]. Nagilactone E, which has been recently studied using the systems biology approach, also inhibits elongation, likely by the same mechanism [39]. The accommodation of aminoacyl-tRNA in the A-site is impeded by the universal antibiotic A201A, which has a nucleoside-like region resembling the CCA-end of tRNA [13, 40].

Some compounds bind to the P-site of the PTC. Among those are two universal inhibitors of the peptidyl transferase reaction – bactobolin A (isocoumarin derivative) [41, 42] and blasticidin S (nucleoside antibiotic) [14, 17, 43, 44]. Interestingly, in bacteria, blasticidin S primarily inhibits translation termination rather than elongation [45], but in eukaryotes, its effect on termination is negligible [46]. A number of insufficiently studied blasticidin-like nucleoside antibiotics, such as anthelmycin (hikizimycin), gougerotin, amicetin, bamicetin, and others [47-49], also weaken aminoacyl-tRNA binding and prevent transpeptidation [43].

Another nucleoside analog interacting with the PTC and affecting ligand binding and accommodation is sparsomycin [17, 18]. The structure of its complex with the eukaryotic ribosome is not yet available, but its interaction with the large ribosomal subunit of archaea has been studied [44]. Based on these structural data, it was suggested that sparsomycin forms multiple contacts with the CCA-end of tRNA in the P-site, while simultaneously preventing the binding of aminoacyl-tRNA to the A-site.

It should be noted that because of the lack of structural and functional data, it is often impossible to unambiguously determine whether the mechanism of action of a particular PTC inhibitor is associated with impaired binding or accommodation of ligands or with conformational rearrangements of the PTC itself (resulting in ineffective catalysis). Therefore, it is uncommon to classify PTC inhibitors further based on a particular stage they block. The situation is further complicated by the recently discovered amino acid specificity of PTC inhibitors. For example, structural data suggest that harringtonine and its derivatives, as well as trichothecene mycotoxins, interfere with the aminoacyl-tRNA entry or at least with the aminoacyl residue accommodation in the A-site [14, 26]. However, the data of the toeprinting assay and ribosome profiling suggest [50-52] that these compounds allow a few elongation cycles to be successfully performed before the ribosome stops at a certain position, which is determined by the amino acid residue at the C-terminus of the peptidyl moiety of the P-site ligand. It remains unclear how the ribosome can synthesize a polypeptide fragment several amino acids long, while its PTC is occupied by a large antibiotic molecule, and why some amino acids can be incorporated successfully, yet the synthesis is blocked on others. The amino acid specificity of PTC inhibitors was first documented in 2013 for harringtonine [50]; it has been shown by the toeprinting technique that this drug arrests the translating ribosome only when the last amino acid attached to the P-site tRNA is lysine, arginine, or tyrosine. Later, the tolerance to the incorporation of certain amino acids into the growing peptide and sensitivity to the others was revealed for many classic PTC inhibitors, including anisomycin, sparsomycin, blasticidin S, and a number of trichothecene mycotoxins [51]. The same phenomenon was observed for some antibiotics blocking the PTC of the bacterial ribosome [53]. However, in the latter case, the specificity was determined by the amino acid residue preceding the one located in the P-site: translation was stopped mainly by alanine (and to a lesser extent, by serine and threonine) in position -1 of the peptidyl-tRNA. This phenomenon changes our understanding of the action mechanism of PTC inhibitors and requires further investigation [54].

*Inhibitors blocking the polypeptide exit tunnel*. The selectivity for the sequence of the nascent peptide is especially pronounced in the case of inhibitors that bind in the ribosome PET. Such drugs are common among the compounds targeting bacterial ribosomes (macrolide antibiotics being a classic example) [55, 56]. Interestingly, macrolides not only hinder progression of the nascent peptide, but also inhibit the PTC. When bound in the ribosomal tunnel, macrolides allosterically affect other regions of the ribosome, in particular, induce conformational rearrangements in the PTC [57]. Some anti-bacterial macrolides can bind to the large subunit of the archaeal ribosome (approximately to the same site as in the bacterial one [58-60]); however, none of them is currently known to interact in the same way with the eukaryotic ribosome [56]. Thus, 13-deoxytedanolide, a non-canonical macrolide targeting eukaryotic 60S subunit [61], binds at a completely different site (see below).

However, small-molecule drugs blocking or altering the peptide tunnel of the eukaryotic ribosome have recently been found among other classes of chemical compounds (figure, e.3). Two recently discovered inhibitors should be mentioned: PF-06446846 and PF-06378503. These unusual drugs exhibit an unprecedentedly high selectivity toward the peptide sequence, so they only block the synthesis of a few proteins of the entire human proteome [62, 63]. A structural study revealed that PF-06446846 binds within the PET [64] and induces ribosome stalling in the intermediate state of translocation due to the altered path of the nascent peptide. Even more recently, another type of eukaryotic translation inhibitors blocking the peptide tunnel was discovered – aromatic polyketides. The binding of tetracenomycin X to the PET in the human ribosome was shown by structural methods and its activity was confirmed by experiments with reporter mRNAs both *in vitro* and in cultured cells [65]. Interactions of tetracyclines Col-3 and doxycycline with the tunnel were studied biochemically [66]. The activity of some other tetracyclines, e.g., tigecycline [67, 68] and minocycline [69, 70], in eukaryotic systems has also been reported, although their binding sites remain unknown. Minocycline can be used for the treatment of autoimmune disorders, neuropathies, and viral infections and has a geroprotective potential. At the same time, the classic antibiotic tetracycline (Tet), which is widely used in medicine as an antibacterial drug, does not block translation in the eukaryotic system and binds to the bacterial ribosome at a completely different site than tetracenomycin X, doxycycline, and Col-3 [7].

*Translocation inhibitors.* Compounds blocking the ribosome at the translocation stage represent a significant portion of eukaryotic ribosome inhibitors (figure, e.6) and utilize various mechanisms of action. The classic eukaryote-specific inhibitor cycloheximide (also known as actidione or naramycin A) occupies the E-site of the 60S subunit and prevents translocation of deacylated tRNA from the P-site [14, 17, 25, 71-75], although alternative mechanisms of its action have also been proposed [34, 76]. Cycloheximide is widely used for the protein half-life assay, stabilization of elongation complexes for the polysome profile analysis, and high-throughput analysis of gene expression by ribosome profiling and translating ribosome affinity purification (TRAP) approaches [77]. It belongs to a group of chemicals called glutarimides, which also includes a number of less-studied translation inhibitors (e.g., streptimidone, actiphenol, acetoxycycloheximide, streptovitacin, isomigrastatin, and others [78-80]). Another glutarimide is lactimidomycin [34, 81], which binds to the same place in the E-site as cycloheximide, but has an additional lactone ring that hinders accommodation of its entire molecule [14]. Unlike cycloheximide, lactimidomycin cannot bind to the actively translating ribosomes, so its addition to the cells leads to the polysome disassembly [34]. This feature of lactimidomycin is exploited in the ribosome profiling assay to map initiation codons [82] (similarly to the previously described harringtonine). The inability of lactimidomycin to displace tRNA from the E-site is related to the slow accommodation of its large side radical [14]. The explanation can probably be applied to all the above cases when elongation inhibitors are inactive toward the ribosomes that have already been engaged in translation in a polysome but successfully interact with tRNA-free ribosomal complexes.

Lissoclimides (in particular, chlorolissoclimide and C45) isolated from sea molluscs bind to almost the same site on the 60S subunit as glutarimide antibiotics. There is also a small degree of structural similarity, so these two classes of antibiotics might have a similar mechanism of action [83-85]. The same site is also targeted by another translation inhibitor with a completely different chemical structure, phyllanthoside [14]. The exact mechanism of its action remains unclear [35], but most likely, phyllanthoside inhibits translocation. The unique property of this drug is presumably formation of a covalent bond with the E-site resulting in its irreversible damage.

It is possible that some polyketides also bind at the same site, e.g., pederins (pederin, theopederins, psymberin, onnamide A, mycalamides, etc.) produced by symbionts of poisonous beetles and marine invertebrates [17, 86, 87], although reliable structural data have been obtained only for one of them, mycalamide A [60]. All these compounds inhibit translocation [17, 87, 88]. Two polyketides of another group, macrolides tedanolide and 13-deoxytedanolide, also block translocation by binding to the same location in the E-site as pederins [61, 89]. Surprisingly, structurally similar myriaporones [90] suppress elongation by phosphorylation of the elongation factor eEF2, rather than by direct binding to the ribosome [91, 92].

Eukaryote-specific inhibitors emetine and related cryptopleurine, as well as the universal antibiotics amicoumacin A and pactamycin, interact with the tRNA-binding region in the E-site, only on the small ribosomal subunit in this case [14, 93-97]. The elucidation of their action mechanism is complicated by the lack of structural data on their complexes with the eukaryotic ribosome in the presence of ligands. The ability of emetine and cryptopleurine to inhibit translocation has been known since the 1970s [17, 25, 98]. Chemically related cephaeline, tylophorine, tylocrebrine, tubulosin, DCB-3503, and YXM-110 also inhibit translocation and presumably bind to the same region of the 40S subunit [95-97, 99-101]. Emetine has been used in medicine for more than a century as an anthelmintic and antiprotozoal (in particular, antiamoebic and antimalarial) medication; recently it was added to the list of potential drugs for combating coronavirus infection caused by SARS-CoV-2 [28].

Amicoumacin A, which affects the same stage of the ribosomal cycle, is considered as a promising anticancer drug [93]. In bacteria, it interacts simultaneously with mRNA and rRNA [102], preventing ribosome movement during the translocation. Since in eukaryotes the transcript is pulled through the ribosome not only during elongation, but also during scanning of the 5′-untranslated region, one would expect amicoumacin A to inhibit the translation initiation. However, functional tests showed that this is not the case: amicoumacin A is a typical elongation inhibitor in eukaryotes as well [93]. Pactamycin interacts with the same region on the eukaryotic ribosome, but its mechanism of action is poorly understood [14, 18, 25]. For a long time, there had been unclear if this drug affects translation initiation or translation elongation (see discussion in [7, 103]). Finally, it was shown that pactamycin inhibits translocation of the bacterial ribosome [104], and our data suggest that it acts via a similar mechanism in eukaryotes [51]. Note that edeine, which is another antibiotic that binds to approximately the same region of the small subunit [14], indeed inhibits initiation (see the corresponding section).

The atypical aminoglycoside hygromycin B blocks translocation by another mechanism. There are no structural data on its interaction with the eukaryotic ribosome. In bacteria, hygromycin B binds to the decoding center (DC) of the small ribosomal subunit, within the helix h44, and induces conformational changes that prevent the movement of mRNA and tRNA from the A-site to the P-site [103, 105]. In eukaryotes, its action mechanism is most likely the same [73, 106]. Other aminoglycosides have a different mechanism of action despite binding to the same site (see the text below); however, at high concentrations, some of them also block translocation. It has been well documented for bacterial ribosomes [107, 108] and can be associated with the anchoring of tRNA in the A-site upon antibiotic interaction with the classic aminoglycoside binding site in the helix h44 or its binding to alternative locations – the large subunit helix H69 or other sites [109, 110]. In a eukaryotic system, this translocation block can be detected by the toeprinting assay only at very high concentrations of paromomycin and G418 [51].

In this section, we did not mention the drugs (for example, sordarin) that block translocation by suppressing the activity of the elongation factor eEF2. As these inhibitors do not affect the ribosome function directly, they will be discussed in a separate section.

*Drugs inducing decoding errors.* A separate class of inhibitors reduces translation fidelity by causing errors in the incorporation of amino acids by the ribosome (figure, e.4). A classic example of this type is the broad-spectrum aminoglycoside antibiotics [111]. Their main binding site on the eukaryotic ribosome is the helix h44 in the DC of the small subunit [14, 110]. The binding stabilizes the DC conformation that is normally adopted only in the presence of the cognate aminoacyl-tRNA in the A-site [7]. This makes transpeptidation possible even when the A-site ligand does match the codon, resulting in the incorporation of a wrong amino acid. Aminoglycosides also induce stop codon readthrough, which makes them promising agents in the therapy of diseases caused by nonsense mutations (see below).

The highest activity against eukaryotic ribosomes is demonstrated by aminoglycosides with the 4,6- or 4,5-disubstituted 2-deoxystreptamine (2-DOS) ring, such as geneticin (G418, widely used for genetic selection in eukaryotic cell cultures) and less active paromomycin, lividomycins, gentamicins, and amikacin [112-117]. Less toxic analogues of G418 and paromomycin (NB50, NB54, NB74, NB84, NB124, NB156, NB157 and others) are also highly active (for details, see [118, 119]), as well as the new promising compound TC007 [110, 120]. The similarities in the chemical structure of aminoglycosides sometimes lead to confusion (as it happened with gentamicin B1 [121]). However, most other known antibiotics of this type are presumably bacteriospecific due to the structural features of the helix h44 in the eukaryotic DC [14, 110, 122, 123]. However, this does not make them safe for eukaryotic cells, as they can suppress mitochondrial protein synthesis and cause severe side effects (primarily nephro- and ototoxicity), which limits their use as antibacterial drugs [124, 125]. As mentioned above, some aminoglycosides also inhibit translocation at elevated concentrations.

*Other mechanisms of elongation failure*. The universal inhibitor puromycin has a unique mechanism of action: it is a molecular mimetic of the aminoacylated CCA-end of tRNA. After entering the A-site, it causes a premature, factor-free termination of polypeptide synthesis [7, 11]. The activity of puromycin is well studied; its fluorescent and biotin derivatives are widely used for the visualization and quantitative analysis of newly synthesized proteins by many modern techniques such as PUNCH-P, SUnSET, Puro-PLA, RiboLace, and RPM, as well as for mRNA display [126]. Treating the cells with puromycin in a combination with cycloheximide leads to the accumulation of ribosomes exclusively on start codons, which facilitates their identification by ribosome profiling [127]. On the other hand, the combined effect of puromycin and other antibiotics depends on the ratio and concentrations of these compounds and cannot always be predicted, which may lead to artifacts [128, 129]. The activity of puromycin is unique; for example, structurally similar antibiotic A201A (see above) does not act as a peptide bond acceptor and only inhibits the peptidyl transferase reaction [13, 40].

Among the bacterial translation inhibitors, there is a group of antibiotics that interact with the GAC of the large ribosomal subunit – the binding site for translational GTPases – and disrupt the functioning of these proteins. This group includes orthosomycins and thiopeptides (evernimicin, thiostrepton, micrococcin and others) that impede accommodation of translation factors on the ribosome [7]. In eukaryotes, the only currently known inhibitor of this type is the antimalarial drug mefloquine (figure, e.2). It binds to the ribosomal protein uL13 and the ES13 region of the 28S rRNA in the vicinity of the GAC [130]. Although its binding site is somewhat different from that of orthosomycins and thiopeptides, mefloquine most likely acts in a similar manner.

GAC is also targeted by plant, fungal and bacterial toxins called ribosome-inactivating proteins and ribotoxins, which cause depurination or cleavage of 28S rRNA at a specific position in the sarcin-ricin loop [131, 132]. However, since these are high-molecular-weight inhibitors, their description is beyond the scope of this review. Besides, the binding of translational GTPases to the GAC is affected by the compounds directly interacting with these factors (described in one of the next sections).

**Ribosome-targeting initiation inhibitors.** The universal inhibitor edeine has an unusual mechanism of action (figure, i.6) [18]. It binds to the 40S subunit in the E-site [14]; however, unlike the above-described pactamycin, emetine, and other translocation inhibitors, it interferes with the recognition of the start codon during scanning (see discussion in [133, 134]). Most likely, edeine interferes with the binding or accommodation of the initiator Met-tRNA_i_ in the P-site, as has been shown for bacteria [7]. It is believed that at adequate concentrations, it does not interfere with the elongation and therefore can be used to analyze the mechanism of translation initiation, although this is sometimes questioned (see review in [135]). The use of edeine in the studies of translation initiation is complicated by the fact that mammalian cells are usually impermeable to this drug (at least to its most common form, edeine A1) [136].

2-(4-Methyl-1,6-dinitroanilino)-N-methyl propionamide (MDMP) affects the final stage of translation initiation, 60S subunit joining (figure, i.9), without interfering with other stages of the translation cycle [137-140]. It presumably targets the ribosome directly [141], but the details of its binding and the mechanism of action are still a mystery. It is also possible that mefloquine (elongation inhibitor discussed in the previous section) may also act at the stage of subunit joining, as it binds to the ribosome in the region shared by the elongation factors and eIF5B, the initiation factor promoting this stage (figure, i.7). The same stage of translation initiation in bacteria is inhibited by the mefloquine functional analogs, thiopeptides, which prevent the binding of IF2 (eIF5B ortholog) [7, 8].

More recently, targeted screening identified a group of compounds that interfere with the 60S subunit binding of initiation factor eIF6 (figure, i.8). These compounds are eIFsixty-1 (clofazimine), eIFsixty-4, and eIFsixty-6 (eIFsixty-4 exhibits the most pronounced effect on the translation and cell growth) [142]. eIF6 is involved mostly in the preparation of newly synthesized ribosomes for the first round of translation after their export from the nucleus, but it may also take part in the regular translation cycle [143]. Unfortunately, due to the lack of structural data, it is unknown whether these compounds target the 60S subunit or the factor itself.

**Ribosome inhibitors affecting termination.** Small-molecule drugs affecting translation termination can be potentially used in the treatment of diseases associated with the nonsense mutations in clinically relevant genes. However, very few specific termination inhibitors with a well-characterized mechanism of action are known (figure, t.2). Although reported to specifically block termination in bacteria [45], blasticidin S primarily affects the elongation stage in eukaryotes [46]. Another antibiotic, apidaecin (insect antimicrobial peptide), interacts with the bacterial ribosome and arrests translation at the stop codon [144]; however, there is no information on its activity in the eukaryotic systems. There is evidence that the anticancer agent girodazole (also known as giroline or RP 49532A) specifically inhibits termination by interacting with the E-site of the 60S subunit and blocking the release of the nascent peptide [89, 145, 146]. Unfortunately, its high toxicity prevents its clinical use [147], so the investigations of this compound have been dropped.

There are many more chemicals that are known to affect the stage preceding the release of the polypeptide, i.e., recognition of the stop codon, thus causing the stop codon readthrough (figure, t.1). Effective and non-toxic readthrough inducers could be widely used in medicine, as more than 10% of hereditary diseases are associated with the nonsense mutations in functionally important genes, while premature stop codons in tumor suppressors are often observed in cancer [148, 149]. Nonsense suppression therapy is aimed to increase the frequency of aberrant amino acid incorporation at the stop codon instead of hydrolysis of the peptidyl-tRNA [150, 151].

The best characterized inhibitors of this type are aminoglycosides (see above), in particular, G418, paromomycin, and gentamicin X2. A decrease in the decoding accuracy caused by these inhibitors leads to the impaired stop codon recognition [110, 111, 113, 115, 152]. Some aminoglycosides (such as G418), when taken at certain concentrations, can induce readthrough without a significant decrease in the overall fidelity of protein synthesis or dramatic effect on the gene expression [113, 115, 117]. Much efforts have been made to develop synthetic aminoglycoside derivatives that would increase the readthrough rate without exhibiting toxicity. An example of such compound is NB124 [114, 119].

However, it is likely impossible to completely eliminate the side effects of aminoglycoside therapy, as the long-term use of these compounds is associated with the risk of nephro- and ototoxicity [111, 153-155]. Therefore, great efforts are directed to finding non-aminoglycoside readthrough-inducing compounds. The best-known result of this search is ataluren (PTC124), a promising candidate in the treatment of cystic fibrosis and other hereditary diseases caused by nonsense mutations [156]. Unfortunately, its clinical trials have not yet been very successful [157, 158]. Moreover, its activity as a readthrough inducer has been called into question, since ataluren was found to affect the stability of a reporter protein [159]. Beside ataluren, a number of other natural and synthetic non-aminoglycoside compounds were found to induce the stop codon readthrough by a still unknown mechanism: GJ071, GJ072, RTC13, RTC14, BZ16, amlexanox, and others identified in high-throughput biochemical screenings (for review, see [150, 160]). A similar effect is likely to be caused by TCP-1109 [161, 162], a derivative of the antibacterial dipeptide negamycin. The latter binds to the bacterial 30S subunit near the DC and causes decoding errors by interfering with the elongation and termination (see [163] and references therein). The nonsense suppression activity in the eukaryotic system was also shown for doxorubicin [152].

Surprisingly, several compounds have been found recently that dramatically enhance the effect of aminoglycosides on the translation termination. The phthalimide derivative CDX5-1, as well as the already mentioned mefloquine (and a number of other quinine derivatives), increase the efficiency of the G418-induced stop codon readthrough by two orders of magnitude [164, 165]. Such combination therapy might allow the use of low concentrations of aminoglycoside to ensure formation of sufficient amounts of full-length proteins encoded by genes with nonsense mutations without accompanying side effects.

**Ribosome recycling inhibitors.** The last stage of the translation cycle is ribosome recycling, which involves ribosomes release after peptidyl-tRNA hydrolysis at the stop codon [166]. Ribosome recycling factors and the underlying mechanisms differ between bacteria and eukaryotes [167]. No chemicals that selectively inhibit this stage have been found yet. However, ribosome recycling in bacteria is affected by aminoglycosides [168]. The structural basis of this activity [109] implies that these compounds might also affect eukaryotic ribosomes. Indeed, paromomycin, neomycin, and hygromycin have been shown to inhibit the dissociation of yeast ribosomes after termination (figure, r.1) [169, 170]. Translocation inhibitors, such as cycloheximide and lactimidomycin, have a similar effect [169, 170]. In addition, compounds suppressing the working cycle of the eEF2 translocase (see the text below) can have some effect on the dissociation of ribosomal subunits in yeast [169, 171]. This is somewhat surprising, however, as it is commonly believed that the involvement of translocase is a specific feature of the bacterial, rather than eukaryotic, type of ribosome recycling [167]. Thus, it cannot be ruled out that some of these observations are associated with the experimental system used by the authors to study ribosome recycling in yeast [169].

## INHIBITORS OF EUKARYOTIC TRANSLATION FACTORS

In this section, we describe inhibitors that bind to translation factors and affect their activity (Table 2). The binding can occur both in solution and on the ribosome during the translation cycle. In the latter case, the drugs can contact both the translation factor and the ribosomal components, but we nevertheless decided to describe them in a separate section.

**Table 2.** Inhibitors of translation factors and ARSases

Name	Class, group of chemical substances	Specificity (B, A, E)	Target	Stage of the translation cycle	Mechanism of action
Aurintricarboxylic acid	triphenylmethane	B, A, E	40S? tRNA? mRNA?	I (E)	inhibits eIF2-GTP-Met-tRNA_i_ complex formation (HC inhibits mRNA and tRNA binding to the ribosome)
Pyrocatechol violet	– // –	B? A? E	– // –	I (E)	– // –
Gallin	xanthene-like, gallin/fluorescein analog	B? A? E	– // –	I (E)	– // –
Gallein	– // –	E?	– // –	I?	– // –
NSC 119893	– // –	E?	eIF2-GTP-Met-tRNA_i_?	I	inhibits eIF2-GTP-Met-tRNA_i_ complex formation
NSC 119889	– // –	E?	– // –	I	– // –
Showdomycin	uridine analog	B, A, E	eIF2, eEF2?	I, E	– // –, eEF2 inhibitor?
Didemnin B	cyclic peptide	E	eEF1A	E	prevents eEF1A-GDP dissociation from the ribosome
Aplidine/plitidipsin/dehydrodidemnin B	– // –	E	eEF1A	E	– // –
Nannocystin A	– // –	E	eEF1A	E	– // –
Cytotrienin A	– // –	E	eEF1A	E	– // –
Tamandarin A	– // –	E	eEF1A	E	– // –
Ansatrienin A/mycotrienin I	cyclic peptide, ansamycin	E	eEF1A	E	– // –
Trienomycin A	– // –	E	eEF1A?	E	– // –
Monoenomycin	– // –	?	eEF1A?	E?	– // –
Trienomycin J	– // –	?	eEF1A?	E?	– // –
Trierixin	– // –	E	eEF1A?	E	– // –
Quinotrierixin	– // –	E	eEF1A?	E	– // –
Ternatin	diterpene alkaloid	E	eEF1A	E	– // –
Tosylphenylalanylchloromethane	chloromethyl ketone	B, A, E?	eEF1A?	E	– // –
Bouvardin	cyclic peptide, bouvardin group	E	eEF2	E	prevents eEF2 GDP dissociation from the ribosome
SVC112	– // –	E	eEF2?		– // –
RA-VII	– // –	E	eEF2?	E	– // –
DDD107498	quinoline derivative	E*	eEF2	E	– // –
Sordarin	cyclic diterpene glycoside, sordarin analog	E*	eEF2	E	– // –
GM193663	– // –	E*	eEF2	E	– // –
GR135402	– // –	E*	eEF2	E	– // –
Moriniafungin	– // –	E*	eEF2	E	– // –
DAO/dihydroarmillylorsellinate	polyketide, sesquiterpene	E	eEF2		– // –
Arnamial	– // –	E	eEF2		– // –
Fusidic acid	steroid	BA(E)	(eEF2)	E	– // – ? (HC)
Allolaurinterol	sesquiterpene	E	eIF4A	I	inhibits eIF4A ATPase activity
Elisabatin A	– // –	E	eIF4A	I	– // –
Rocaglamide A/Roc A	rocaglate	E	eIF4A	I	inhibits eIF4A helicase activity and eIF4F binding to mRNA
Silvestrol	– // –	E	eIF4A	I	inhibits eIF4A helicase activity
Pateamine A	macrodiolide	E	eIF4A	I	impedes eIF4A binding to eIF4G
Hippuristanol	steroid	E	eIF4A	I	allosteric inhibitor of eIF4A
Ribavirin	m^7^G analog	E	eIF4E?	I	competes with m^7^G-cap for eIF4E binding?
4EGI-1		E	eIF4E-eIF4G	I	impedes eIF4E binding to eIF4G
4E1RCat		E	eIF4E-eIF4G	I	– // –
4E2RCat		E	eIF4E-eIF4G	I	– // –
Gephyronic acid	polyketide	E	(eIF2)	I	binds to eIF2 and affects its activity?
CM16	beta-carboline	E	eIF1AX, eIF3	I	inhibits eIF1AX and eIF3?
Ochratoxin A	isocoumarin	B, A, E	Phe-tRNA synthetase	E	inhibits Phe-tRNA synthetase
Borrelidin	polyketide	B, A, E	Thr-tRNA synthetase	E	inhibits Thr-tRNA synthetase
Reveromycin A	– // –	E	Ile-tRNA synthetase	E	inhibits Ile-tRNA synthetase
Spirofungin A	– // –	E	– // –	E	– // –
Furanomycin	Ile analog	B? A? E	– // –		– // –
Methionine sulfamide	Met analog	B, A, E	Met-tRNA synthetase	E	inhibits Met-tRNA synthetase
Methionyl adenylate	– // –	B, A, E	– // –	E	– // –
Methionine hydroxamate 20	– // –	B, A, E	– // –	E	– // –
Ethionine	– // –	B, A, E	– // –	I, E	– // –
Tavaborole/AN2690	oxaborol	E	Leu-tRNA synthetase	E	inhibits Leu-tRNA synthetase
Histidinol	His analog	B, A, E	His-tRNA synthetase	E	inhibits His-tRNA synthetase and His biosynthesis
Phosmidosine	nucleoside amidophosphite	E	Pro-tRNA synthetase?	E	inhibits Pro-tRNA synthetase
Febrifugine	quinazolinone alkaloid, febrifugine group	?	– // –	E	– // –
Halofuginone	– // –	?	– // –	E	– // –
Purpuromycin	polyketide	B, A, E	tRNA	E	binds any tRNA, prevents its aminoacylation
GC7	spermidine analog	B, A, E	DHPS/eIF5A	E	inhibits hypusine synthesis necessary for eIF5A activity
Semapimod/CNI1493	anilide	E	– // –	E	– // –
Deoxyspergualin/gusperimus		E	– // –	E	– // –
DHSI-15		E	– // –	E	– // –
Ciclopirox/loprox	pyridone derivative	E	DOHH/eIF5A	E	inhibits hypusine synthesis necessary for eIF5A activity (iron chelator)
Deferiprone	– // –	E	– // –	E	– // –
Mimosine	– // –	E	– // –	E	– // –

Note. Letter codes and designations – as in Table 1.

**Inhibitors of elongation factors.** A large group of chemically diverse substances, usually derived from marine organisms (both bacteria and eukaryotes), target the eEF1A elongation factor [172, 173]. They bind to a specific site on the protein surface and modulate protein conformational dynamics, which results in the inability of eEF1A to dissociate from the ribosome after GTP hydrolysis (figure, e.2), thus blocking elongation. It is unclear why this particular stage is especially attractive for inhibition, but the same mechanism of action is shared by unrelated compounds, such as cyclic depsipeptides from the didemnin group [174] (didemnins A, B, C, and M [175, 176], aplidine/plitidipsin [177], tamandarins A and B [178]), ansamycins (cytotrienin A [179] and similar trienomycins, trierixin, quinotrierixin and ansatrienins A and B, also called mycotrienins I and II [173, 180]), and the cyclic peptide ternatin [173]. Nannocystin A is another macrocyclic compound with a more complex structure, but the same mechanism of action [181]. All these drugs are, in fact, eukaryote-specific functional analogs of the well-known antibacterial inhibitor kirromycin, which stabilizes the EF1α complex with aminoacyl-tRNA on the ribosome [7].

A distinct group of compounds uses a similar mechanism to inhibit another elongation factor, the eEF2 translocase (figure, e.7). The classic examples are the fungicide sordarin and its numerous derivatives (moriniafungin, GM193663, GR135402, azasordarins, etc.), which target eEF2 in some fungi, but are harmless for human cells [182-185]. Their binding to eEF2 [186, 187] prevents its dissociation from the ribosome and thus freezes the elongation complex in a post-translocational state [188]. The action of sordarin resembles that of the well-known antibacterial antibiotic fusidic acid, although there is a difference in the details of its interaction with the factor (discussed in [188]). Fusidic acid itself is likely unable to specifically inhibit translocation in eukaryotic cells, although at high concentration it may have some effects [189].

The inhibitory activity of sordarin requires diphthamide, an AE-specific, uniquely modified amino acid only found in eEF2 [190]. Interestingly, diphthamide is a target for a large group of bacterial protein toxins (diphtheria toxin and others) that inactivate eEF2 by ADP-ribosylation of this residue. Yet, inhibitors of protein nature are beyond the scope of our review, so we refer interested readers to the publication [191].

The question of whether there is a sordarin analogue that is active in mammalian cells is still open. Most likely, similar mechanism of action can be attributed to the cyclic peptide bouvardin (anticancer drug) and its derivatives (RA-VII, SVC112, etc.) [192, 193]. It had been reported that purpuromycin may act in a similar way [194], but later its activity was linked to the inhibition of aminoacylation (see below).

Recently, the antimalarial drug DDD107498, which is non-toxic to human cells, has been discovered and shown to target eEF2 of the malaria parasite [195]. Interestingly, it contains the same quinoline heterocycle as the above mefloquine. It cannot be ruled out that all quinine-like compounds used for malaria treatment disrupt the interaction of elongation factors with the ribosome [130].

eIF5A is another elongation factor (formerly erroneously believed to be an initiation factor) that can also serve as a target for the inhibitors, more precisely, those that target the synthesis of hypusine, a uniquely modified amino acid residue required for the eIF5A activity [196]. Conversion of the conserved lysine residue to hypusine can be blocked at different stages by a number of compounds, leading to the accumulation of inactive factor. Such inhibitors include GC7, semapimod (CNI1493), deoxyspergualin (gusperimus), DHSI15, ciclopirox, deferiprone, and mimosine (see review in [196]). These compounds, however, are not highly specific. Some of them inactivate other enzymes involved in the metabolism and transport of polyamines and other molecules, while ciclopirox, deferiprone, and mimosine are iron chelators. Mimosine was previously shown to indirectly target another translation factor, eIF3, by specifically downregulating the production of the eIF3a subunit [197].

**Inhibitors of initiation factors.** In contrast to relatively conserved elongation factors, many eukaryotic components of the translation initiation machinery have appeared in the evolution with the emergence of ribosomal scanning and, therefore, are eukaryote-specific. This primarily refers to the eIF4 group of initiation factors, which facilitate mRNA binding to the ribosome and direct scanning [1]. The small cap-binding protein eIF4E, a component of the heterotrimeric eIF4F complex, anchors to the m^7^G-capped 5′-end of mRNA, while its partner, the mRNA-binding factor eIF4G, serves as a platform for the ATP-dependent RNA helicase eIF4A and bridges mRNA with the factors bound to the ribosomal 43S preinitiation complex [1].

Targeted high-throughput screening [198] identified compound 4EGI-1 that binds to eIF4E and allosterically disrupts its association with eIF4G (figure, i.3), while simultaneously enhancing its interaction with the inhibitory protein 4E-BP1 [199, 200]. Thus, 4EGI-1 suppresses cap-dependent mRNA translation with no effect on the transcripts employing non-canonical initiation mechanisms [e.g., viral mRNAs with internal ribosome entry sites (IRESs) or cellular mRNAs with cap-independent translation enhancers (CITEs)] [201]. Another screening identified two more compounds with a similar mechanism of action, 4E1RCat [202] and 4E2RCat [203]. The latter exhibited strong antiviral activity and was able to suppress the propagation of coronaviruses.

There is a hypothesis that some cardiac glycosides (e.g., ouabain) affect translation in a similar way [204]. Transcriptional changes induced by cardiac glycosides strongly resemble those caused by the classic elongation inhibitors (cycloheximide, anisomycin, emetine, etc.) [205]. Moreover, Perne et al. showed suppression of protein synthesis in the cells treated with these substances [206]. However, these effects are most likely secondary or temporary, since the similarity of transcription patterns, strongly pronounced at the 6th hour of exposure, disappeared by 24 h [205]. It is also possible that these drugs inhibit the PI3K/Akt/mTOR signaling pathway [207] (see the text below) or the initiation factor eIF4A [208]. However, in direct experiments in a mammalian cell-free system, cardiac glycosides failed to noticeably inhibit translation of reporter mRNAs (Lashkevich and Dmitriev, personal communication).

eIF4E interaction with the m^7^G cap at the mRNA 5′-end is also very important. Kentsis et al. stated [209] that this interaction can be disrupted by a competitive inhibitor (figure, i.4) – the antiviral drug ribavirin (and its triphosphorylated form), which structurally resembles the m^7^G-cap. This statement was challenged by two separate groups [210, 211], yet the authors of the original study remained unconvinced [212]. Later, ribavirin was shown to suppress the Akt signaling pathway [213], which can explain its effects.

The J. Pelletier group has discovered a number of new inhibitors that target another component of the eIF4F complex – the RNA helicase eIF4A [214, 215]. Hippuristanol, a polyoxygenated steroid, binds and allosterically inhibits eIF4A (figure, i.5) [216], while pateamine A prevents eIF4A interaction with eIF4G (figure, i.2) and increases its RNA binding activity [217, 218]. Rocaglates (including rocaglamide A, silvestrol, and other flavaglines) also suppress the activity of eIF4A, but their mechanism of action is less characterized (figure, i.5) [219-221]. Ribosome profiling revealed that eIF4A inhibition by some of these drugs causes a sequence-specific arrest of the scanning ribosome at the 5′-untranslated region (see discussion in [221]). In the latest study, rocaglates were found to act in a dual fashion: first, they disturb the landing of eIF4F and the initiator complex on the 5′-cap and then inhibit the ribosomal scanning [221]. Numerous derivatives of the eIF4A inhibitors have been obtained and characterized for the use in the anticancer therapy [221, 222]. Recent screening revealed two new, highly specific ATP-competitive inhibitors of eIF4A – elisabatin A and allolaurinterol [223]. There are also a number of drugs (e.g., nucleoside analogs such as hypericin) that target translational RNA helicases (not only eIF4A, but also DDX3) in a less specific manner [214].

Translation initiation is the primary target of aurintricarboxylic acid (ATA) and similar triphenylmethane and xanthene dyes (pyrocatechol violet, gallin, and some others) that are universal inhibitors widely used in early *in vitro* studies of protein synthesis [18, 224, 225]. However, the specificity of their action is questioned, since at higher concentrations, they can also inhibit other translation steps (reviewed in [10]). ATA and similar chemicals are likely to reduce both specific and nonspecific RNA-protein interactions [226, 227], thus inhibiting factor-dependent and non-enzymatic tRNA binding to the ribosome during initiation (figure, i.1) [228]. This relaxed specificity, as well as inability to enter intact mammalian cells [229], have led to the loss of interest in their use for studying eukaryotic translation.

However, in 2004, while searching for new translation inhibitors, several similar xanthene-based compounds, such as gallein and fluorescein derivatives, were discovered that produced an interesting mRNA-specific effect on the translation of reporter transcripts [230]. Their addition to a cell-free system suppressed cap-dependent translation, but had no effect on the protein synthesis directed by the IRES of the hepatitis C virus (HCV). Among other features, this IRES is known to provide the eIF2-independent translation initiation under certain conditions [231, 232]. A more detailed study of compounds NSC 119889 and NSC 119893 (the latter is cell-permeable) showed that they prevent the binding of the initiator Met-tRNA_i_ to eIF2 (figure, i.1) and thereby block the formation of the 43S preinitiation complex [232], an essential intermediate of the canonical translation initiation.

Several other translation factors (e.g., eIF1AX and eIF3 [197, 233]) were also identified as targets for small-molecule inhibitors, but these interactions have not yet been sufficiently studied. Furthermore, factor-mediated functions can also be blocked by the non-hydrolyzable analogs of ribonucleoside triphosphates. Thus, GTP analogs (GMPPNP and GMPPCP) inhibit initiation, elongation, and termination stages, while ATP analogs usually interfere with initiation, ribosome recycling and functioning of (ARSases). However, these inhibitors are obviously nonspecific and, in most cases, cell-impermeable [229].

## INHIBITORS OF AMINOACYL-tRNA SYNTHETASES

Beside translation factors, small chemical compounds can target other auxiliary components of the protein synthesis machinery. Unsurprisingly, inhibitors of ARSases specifically block protein synthesis (Table 2 and figure, e.1). Sulfonamides, hydroxamates, and other derivatives of amino acids and peptides, as well as esters and hydroxamates of aminoacyl adenylates, inhibit the synthesis of the corresponding aminoacyl-tRNAs. For example, L-methioninol, methionyl sulfamide, L-methionyl hydroxamate, and methionyl adenylate derivatives specifically inhibit the synthesis of Met-tRNA [234, 235], while the Trp antagonist 6-fluorotryptophan inhibits amino acid activation in the tryptophanyl adenylate synthesis [236]. There are numerous studies exploring such amino acid derivatives [10], and this field is growing rapidly due to the development of computer-aided drug design [237-239]. In rare cases, amino acid analogs (e.g., ethionine, an S-ethyl analogue of Met) not only inhibit ARSases, but can be also incorporated into proteins, leading to cell death [240].

Most of the above compounds are universal protein synthesis inhibitors and can freely pass into a living cell. However, due to the high similarity to amino acids, they can affect other cellular processes. In addition, their effective concentrations are usually in a relatively high (millimolar) range. However, there are several specific ARSase inhibitors produced by some pathogenic organisms with a much higher affinity for their targets. For example, borrelidin, a product of marine bacteria, is a highly specific inhibitor of Thr-tRNA synthetase [241]; ochratoxin A from mold fungi targets Phe-tRNA synthetase [242]; febrifugine and halofuginone inhibit Pro-tRNA synthetase [243, 244], while tavaborole inhibits Leu-tRNA synthetase [245]. Ile-tRNA synthetase is targeted by spirofungin A [246] and reveromycin A [247, 248], although the effects of the latter might be cell type-specific [249]. Finally, the unusual inhibitor purpuromycin can bind any tRNA and prevent its aminoacylation without affecting the binding of already aminoacylated tRNAs to the elongation factors, ribosome, and other translational components [250], which makes it somewhat special.

Many ARSase inhibitors are of great medical importance, as they have the immunosuppressive activity and are extensively used as antimicrobial, antitumor, and antiparasitic agents [239, 251]. Their effects on the living cell are usually mediated not only by the suppression of protein synthesis, but also by triggering a special type of stress response [252] caused by the accumulation of deacylated tRNAs in the cytoplasm and collisions of translating ribosomes (see below).

## INHIBITORS OF SIGNALING PATHWAYS INVOLVED IN TRANSLATIONAL CONTROL

Like any other complex process in the cell, almost every step of protein biosynthesis is precisely regulated at multiple levels. Eukaryotes have a number of signaling cascades ending in specialized enzymes that modify translational components [253, 254]. These regulatory pathways deserve a separate review, so we will not discuss all of them, but will focus on some components of these cascades serving as targets for protein synthesis inhibitors (Table 3 and figure, s.1-s.9).

**Table 3.** Inhibitors of the general signaling pathways that regulate protein biosynthesis in eukaryotic cells

Rapamycin/sirolimus	macrolide, rapamycin group	FKBP12/mTORC1	I (E)	allosteric mTOR inhibitor (mTORC1 only); activates 4E-BP1 and suppresses cap-dependent translation, primarily 5′-TOP mRNAs
Everolimus	macrolide, rapamycin group (rapalog)	– // –	I (E)	– // –
Temsirolimus	– // –	– // –	I (E)	– // –
Ridaforolimus	– // –	– // –	I (E)	– // –
Torin 1	pyridinonequinoline	mTOR	I (E)	ATP-competitive mTOR inhibitor (both mTORC1 and mTORC2), activates 4E-BP1 etc.
Torin 2	– // –	– // –	I (E)	– // –
Torkinib/PP242	pyrazolopyrimidine	– // –	I (E)	– // –
Sapanisertib/MLN0128/INK128/TAK-228	benzoxazole	– // –	I (E)	– // –
Vistusertib/AZD2014	phenylpyridine, vistusertib group	– // –	I (E)	– // –
AZD8055	– // –	– // –	I (E)	– // –
Dactolisib/NVP-BEZ235	phenylquinoline	PI3K (mTOR)	I (E)	– // –
Voxtalisib/SAR245409/XL765	pyrazolylpyridine	– // –	I (E)	– // –
Samotosilib/LY3023414	imidazoquinoline	– // –	I (E)	– // –
Omipalisib/GSK2126458	quinoline	– // –	I (E)	– // –
Wortmannin	steroid	– // –	I (E)	– // –
LY294002	morpholine derivative	– // –	I (E)	– // –
Bimiralisib/PQR309	pyridinamine	– // –	I (E)	– // –
Gedatolisib/PKI-587/PF-05212384	benzoylpiperidine	– // –	I (E)	– // –
Adavosertib/MK1775	piperazine	GCN2?	I	activates GCN2?, leads to suppression of 5′-TOP mRNA translation
Dabrafenib	sulfanilide	– // –	I	– // –
BTdCPU	N,N′-diaryl urea	HRI	I	activates HRI, induces eIF2 phosphorylation
CCT020312	quinoline	PERK	I	activates PERK, induces eIF2 phosphorylation
MK-28	methylaminopentanamide	– // –	I	– // –
Salubrinal	quinoline, salubrinal group	GADD34/PP1?, CReP/PP1?	I	inhibits eIF2-specific PP1 phosphatase complexes, induces eIF2 phosphorylation
Sal003	– // –	– // –	I	– // –
Okadaic acid	polyketine derivative of C_38_-fatty acid	PP2A	I	inhibits PP2A phosphatase, induces eIF2 phosphorylation
ISRIB	cyclohexylacetamide	eIF2B	I	modulates eIF2B activity, prevents translation inhibition
Myriaporone 3/4	polyketide, tedanolide analogue (see Table 1)	eEF2K?	E	induces eEF2 phosphorylation
Nelfinavir/viracept		– // –	E	– // –
NH125	methylimidazolium iodide	– // –	E	– // –
A-484954	pyrimidine-6-carboxamide	eEF2K	E	eEF2K inhibitor, prevents translation inhibition

Note. For letter codes and designations, see Table 1.

**Inhibitors of mTOR kinase and the PI3K/Akt/mTOR signaling cascade.** A very important signaling pathway is the PI3K/Akt/mTOR regulatory cascade, which integrates signals from insulin and a number of growth factors, as well as from the sensors of nutrient availability [255, 256]. One of the direct substrates of the mTOR kinase is the above-mentioned inhibitor protein 4E-BP1. When phosphorylated, it remains inactive and does not interfere with the functioning of the cap-binding factor eIF4E [256]. But if mTOR is inhibited, 4E-BP1 displaces eIF4G from its complex with eIF4E. This results in a moderate decline in total protein synthesis and a much more severe suppression of translation of a special class of mRNA transcripts with the 5′-terminal oligopyrimidine tract (5′-TOP) [201, 255]. 5′-TOP mRNAs mainly encode components of the translational apparatus (ribosomal proteins, translation factors, etc.) [257, 258], the synthesis of which is especially important for actively proliferating and metabolizing cells, including tumor and stem cells [259]. The activity of this pathway strongly decreases with age [260] and can affect the lifespan [261]. mTOR substrates also include S6 kinases 1/2, which phosphorylate the ribosomal protein eS6 (RPS6), translation initiation factor eIF4B, eIF4A inhibitory protein PDCD4, and, indirectly, eEF2 [253, 254]. All this makes mTOR an attractive target for clinically relevant drugs [259]. By now, many mTOR inhibitors have already been found (see Table 3 for the most commonly used ones). They can be divided into two types: direct ATP-competitive inhibitors that target the active site of the kinase (figure, s.6) and allosteric inhibitors that act indirectly through the FKBP12 protein, a component of the mTORC1 kinase complex (figure, s.7). mTORC1 is mainly responsible for the translation-related branch of the mTOR pathway. Many commonly used drugs, such as torin 1, torin 2, INK128, AZD-8055, OSI-027, WYE-132, Ku0063794, and PP242 [255, 262], are direct mTOR inhibitors, while allosteric inhibitors include the widely-known natural macrolide rapamycin (sirolimus) and its synthetic analogs called rapalogs (everolimus, temsirolimus, and ridaforolimus) [255, 259]. Rapalogs have long been successfully used in anticancer therapy and as immunosuppressants in organ transplantation. There is also a growing interest in mTOR inhibitors as geroprotectors, since they have been shown to increase longevity in a number of animal models [261].

Some compounds known to interfere with the cap-dependent translation target the upstream components of the PI3K/Akt/mTOR cascade rather than mTOR itself (figure, s.5). As we move up the cascade, the effects of the inhibitors expand and increase, while the specificity decreases. Nevertheless, PI3K inhibitors (e.g., wortmannin and LY294002) are often used to suppress the cap-dependent translation. It should be noted, however, that the kinase domains of PI3K and mTOR belong to the same family and thus share common inhibitors [263]. The top hits in a recent screening for the compounds suppressing translation of 5′-TOP mRNAs [264] included inhibitors of each of the PI3K/Akt/mTOR cascade components (and quite unexpectedly, the GCN2 kinase; see below). It is also possible that cardiac glycosides target the mTOR pathway with a certain degree of specificity [207].

However, it should be kept in mind that mTOR has several dozen substrates, including those unrelated to translation. Therefore, the effect of its inhibitors on the protein synthesis is not highly specific. The same can be said about compounds targeting the MAPK cascades (Ras/ERK/RSK and p38MAPK/Mnk1/2), which share some components with the PI3K/Akt/mTOR pathway [253, 254]. Although MAPK signaling regulates the activity of some general translation factors, such as eIF4E, eIF4B, and eEF2, its effects on the cell functions are too broad; besides, there is no full understanding of how these cascades affect protein synthesis in general.

**Modulators of eIF2 phosphorylation.** Protein kinases phosphorylating the initiation factor eIF2 are much more specific. Mammals have four such kinases: GCN2, HRI, PERK, and PKR [265, 266]. eIF2 delivers the initiator Met-tRNA_i_ to the P-site of the 40S subunit and provides scanning and AUG selection [5]. It is a key factor necessary for all eukaryotic mRNAs, except extremely rare cases when the start codon is positioned into the P-site without scanning (discussed in [267]). Phosphorylation of the eIF2 α-subunit prevents protein dissociation from the inactive complex with eIF2B (guanine nucleotide exchange factor) and thus removes it form the active pool. Until recently, it had been believed that eIF2 is the only substrate for these four kinases; now we know that this is not entirely true [265, 268]. Nevertheless, there is no doubt that the main function of these kinases is regulation of protein synthesis. Therefore, in this section, we will discuss compounds modulating their activity.

BTdCPU and other N,N′-diaryl urea derivatives are specific HRI activators (figure, s.2), which are also considered as promising antitumor drugs [269]. CCT020312 and MK-28 are pharmacological activators of PERK (figure, s.1) and can be potentially used in the treatment of neuropathies [270, 271]. Specific activators of GCN2 kinase (figure, s.3) have not yet been sufficiently studied; however, recent screening identified two candidates – small molecules dabrafenib and MK1775 [264]. Interestingly, their addition to the human cells results in the suppression of the 5′-TOP mRNA translation, strongly resembling the effect of mTOR inhibitors (see above). Although the underlying mechanism has not been elucidated, earlier studies suggest a link between GCN2 and 5′-TOP mRNA, mediated by TIA-1/TIAR proteins [272].

Another possibility to dramatically increase the level of eIF2 phosphorylation is inhibition of the eIF2-specific phosphatase complexes. Thus, salubrinal and its water-soluble derivative Sal003 inhibit protein synthesis by inactivating stress-inducible GADD34/PP1 and constitutively active CReP/PP1 complexes (figure, p.4) [273, 274]. An increase in the eIF2 phosphorylation has also been reported when the cells were treated with okadaic acid, an inhibitor of PP2A phosphatase [275]; however, it is unclear if this effect was specific, as eIF2α is most likely a substrate of PP1 rather than PP2A [276]. Nevertheless, both phosphatases play an important role in the translational control [277] and can be promising targets in the inhibition of protein synthesis.

Not only activators, but also many suppressors of the four kinases have been identified. They, however, have little or no effect on the protein synthesis level in living cell under normal conditions. At the same time, they prevent a decline in the translation under stress conditions. Since the transient translational block is an important part of the stress response, its disruption may have detrimental consequences for the stressed cell. Although these specific effects are important in the antiviral therapy, treatment of neurological disorders, and for promoting the effects of tumor chemotherapeutic drugs in oncology, they are beyond the scope of our review. Those interested in this topic can be referred to the review by Joshi et al. [268].

Nevertheless, we would like to mention the small molecule *trans*-ISRIB, which has a very unusual mechanism of action [278]. It binds to eIF2B and, up to certain limits, maintains its GDP/GTP-exchanging activity toward phosphorylated eIF2 (see details in [279]). As protein synthesis in the brain is necessary for the short-term memory consolidation into the long-term memory, ISRIB can promote memory and enhance cognitive abilities in animals [278].

When discussing the inhibitory effect of eIF2 phosphorylation, we should also mention several chemicals that are often used by researchers for its indirect induction. These include tunicamycin (inhibitor of protein glycosylation causing endoplasmic reticulum stress), thapsigargin (inducer of Ca^2+^ release from the intracellular stores), and dithiothreitol (a thiol reducing agent, which triggers unfolded protein response, or UPR). All of them indirectly activate PERK. Other widely used chemicals are sodium arsenite (selectively targets SH-groups in some proteins and triggers HRI activation), nonspecific oxidants such as hydrogen peroxide (also probably induces HRI), long double-stranded RNAs (PKR activators), some of the already mentioned amino acids analog, and ARSase inhibitors (indirectly activate GCN2). eIF2 phosphorylation is also induced by various blockers of the mitochondrial respiratory chain, ATP synthase, and glycolysis (e.g., myxothiazole, 2-deoxyglucose, oligomycin) and other inhibitors of cell energy metabolism. However, we will not discuss them in detail, since their action is nonspecific, while translation inhibition is only one of the components of the integrated stress response (ISR) that is triggered in cells by the action of stress factors [265, 266].

**Inducers of eEF2 phosphorylation.** The eEF2 elongation factor is inhibited by phosphorylation. Its activity is controlled by several kinases, but the main one is the specialized kinase eEF2K. Being a substrate of S6K and mTOR, it is negatively regulated by the PI3K/Akt/mTOR cascade. When this cascade is suppressed, eEF2K is activated, while eEF2 is partially suppressed [253]. eEF2K inhibitors produce no noticeable effect on the cells under normal conditions (see [280]), whereas eEF2K activators can significantly reduce the efficiency of translation (figure, s.8). For example, the polyketide myriaporone 3/4, which resembles the above-described ribosome inhibitors tedanolide and 13-deoxytedanolide [90], induces eEF2 phosphorylation [92] and thus negatively affects translation [91]. The antiviral drug nelfinavir [281] and compound NH125 (previously mistakenly thought to be an eEF2K inhibitor) have the same effect, although in the latter case, its association with eEF2K is not obvious [269, 282]. Modulators of the eEF2K activity are gaining an increasing attention of researchers due to the emerging role of this kinase in the development of depression, epilepsy, and neurodegenerative disorders [283].

AMPK is another kinase involved in the regulation of protein biosynthesis that should be mentioned in the context of eEF2 phosphorylation. It is a sensor of cell energy status and, when activated, contributes to the reduction of the elongation rate by signaling to eEF2K and phosphorylation of eEF2 [253]. There are many small molecules activating AMPK (figure, s.9) either indirectly via ATP depletion (e.g., 2-deoxyglucose, oligomycin, and the antidiabetic drug metformin) or directly. The latter include A-769622, benzimidazole derivative 991, and the most famous AMPK activator AICAR used by unscrupulous athletes for doping [284]. All these compounds eventually induce eEF2 phosphorylation, thereby decreasing the translation efficiency (discussed in detail in [285]). Needless to say, similar to the effects of inhibitors of the mTOR pathway (which shares some substrates with AMPK), the effects of AMPK activators on the cells are broad and not limited to the suppression of protein synthesis [254].

## RIBOTOXIC STRESS AND TRANSLATION-RELATED STRESS RESPONSES

When studying the effects of translation inhibitors, one cannot ignore the response that occurs in cells upon partial or complete cessation of protein synthesis. Eukaryotes have several mechanisms that monitor the status of the general translational activity, including availability of amino acids, fidelity of the co-translational folding, and correct addressing of protein products. They are also involved in the translation quality control of individual transcripts by each ribosome [6, 286]. At the end of the last century, it was shown that mammalian cells exposed to anisomycin or some other elongation inhibitors activate a special program called the ribotoxic stress response [287]. Interestingly, despite the same level of translation suppression, some inhibitors (anisomycin, deoxynivalenol) trigger a significant activation of the JNK/p38MAPK signaling pathway, leading to the rRNA cleavage and cell death, while others (for example, pactamycin) completely lack this ability [287-290]. It was further revealed that even chemically similar substances with the same mechanism of action might differ fundamentally in the stress level they cause. For example, diacetylanisomycin, a close derivative of anisomycin, does not activate the stress response at all [288]. Trichothecene mycotoxins with different side radicals vary dramatically in the ability to induce the response [288, 291, 292], although all of them inhibit the PTC. Theopederin, onnamide A, and 13-deoxytedanolide bind to different sites on the ribosome and inhibit translocation; however, they activate ribotoxic stress similarly to anisomycin [86, 293]. On the other hand, the translocation inhibitor cycloheximide is a relatively weak stress inducer [287, 294], while lactimidomycin (another glutarimide) strongly activates p38MAPK [294]. Cytotrienin A, while targeting eEF1A, also causes severe ribotoxic stress, [295], as well as some aminoglycosides, the ototoxicity of which may be associated, in particular, with the induction of this type of stress [296].

Until recently, the mechanism for the induction of the ribotoxic stress response had remained a mystery, although the intermediate components of the cascade – JNK and p38MAPK kinases – were identified in the very first study on this topic [287]. Later, PKR and HCK kinases were also named as candidates for the mediators or primary inducers [287-290]. Recently, it was found that some elongation inhibitors, normally inducing the ribotoxic stress response, fail to trigger it when used in higher concentrations [67], suggesting that this phenomenon is based on the activation of certain signaling cascades by the two or three colliding ribosomes. When elongation is partially blocked by a drug, these collisions occur much more frequently, resulting in the induction of generalized cell response. However, if all ribosomes are arrested simultaneously, such collisions become impossible. This hypothesis was brilliantly confirmed in a recent study by the R. Green group [67]. The authors showed that the collided ribosomes orchestrate three different molecular pathways that have been previously considered independent. In the case of a single collision, the mechanisms of ribosomal quality control (RQC) are induced [6, 286], leading to the disassembly of the stalled elongation complex without triggering the general response. As the number of such events increases (for example, during amino acid starvation), the binding of GCN1 and GCN20 proteins and the MAP3K cascade kinase ZAK to the colliding ribosomes activates GCN2 kinase, which phosphorylates eIF2 (see above). If the number of collision sharply increases (e.g., upon antibiotic treatment or exposure to ultraviolet radiation), ribosome-bound ZAK activates the JNK/p38MAPK signaling cascade and triggers the ribotoxic stress response [67, 294]. Indeed, it has long been known that this type of stress can be partially suppressed by the small-molecule inhibitors DHP-2, sorafenib, and nilotinib, which target ZAK [297-299]. These findings might also explain recent observation that the cell response to ARSase inhibitors (see above) is dissimilar to that induced by regular amino acid starvation [252]. The response to deacylated tRNA and frequent ribosome collisions is based on the activation of GCN2 and/or MAPK stress kinases and occurs via different scenario than the starvation response, which “senses” amino acids availability through a cascade of interactions involving mTOR kinase [300].

## CONCLUSION

Protein biosynthesis is one of the major metabolic processes that is crucial for maintaining all body functions. In actively proliferating cells, it consumes a significant portion of their energy and resources. Disruption of protein biosynthesis leads to an inevitable arrest of cell division and death. It is not surprising that translation is the “Achilles heel” of tumor cells and actively propagating viruses [136, 203, 259, 301, 302]. The development of small-molecule inhibitors for manipulating protein synthesis is very important in the anticancer and immunosuppressive therapy, treatment of hereditary, viral and fungal diseases, neurology, parasitology and geriatrics, solving problems of lifespan extension, as well as agriculture, veterinary, and other fields [8, 27, 132, 148, 151, 155, 214, 259, 261, 301, 302]. However, their clinical use is still limited due to the cytotoxicity-related side effects. Rapid development of high-throughput screening techniques [303], as well as machine learning [304] and computer modeling, in combination with modern methods of structural analysis and chemical synthesis [84, 237-239] has given hope for rapid progress in the development of new drug derivatives with improved therapeutic properties. The impact of systems biology approaches on the search for and characterization of new inhibitors will also undoubtedly increase. For example, comparison of transcription patterns helps to determine the mechanisms of action of newly discovered and previously known compounds [39, 205]. In our opinion, another underestimated approach is the combination therapy with different types of inhibitors [164, 165]. Finally, due to the variety of action mechanisms, many of the described compounds can be widely used not only in practice, but also in basic research to study the principles of protein synthesis and translational control [4, 16, 24, 82, 127, 134, 267, 305].

In this review, we attempted to describe the major classes of small-molecule inhibitors of eukaryotic translation. However, the number of currently known drugs with this activity, even those with characterized mechanism of action, reaches several hundred, so it was impossible to review all of them in one article. Besides, this list is constantly growing. Therefore, we have developed a constantly updated Eukaryotic Protein Synthesis Inhibiting Compounds (EuPSIC) database that contains additional information that can be used to facilitate machine processing, such as PubChem numbers and literature references with PubMed IDs. The database can be found at http://eupsic.belozersky.msu.ru/.

## Abbreviations

5′-TOP: 5′-terminal oligopyrimidine tract
ARSase: aminoacyl-tRNA synthetase
DC: decoding center
GAC: GTPase-activating center
PET: ribosomal peptide tunnel
PTC: peptidyl transferase center
